# Renal mucinous tubular and spindle cell carcinoma: a report of 8 cases and review of the literature

**DOI:** 10.1186/1746-1596-8-206

**Published:** 2013-12-11

**Authors:** Xiao-rong Wu, Yong-hui Chen, Jian-jun Sha, Ling Zhao, Ji-wei Huang, Juan-jie Bo, Dong-ming Liu, Yi-ran Huang

**Affiliations:** 1 Department of Urology, Ren Ji Hospital, School of Medicine, Shanghai Jiao Tong University, Shanghai 200001, China; 2 Department of pathology, Ren Ji Hospital, School of Medicine, Shanghai Jiao Tong University, Shanghai 200001, China

**Keywords:** Renal cell carcinoma, Mucinous tubular and spindle cell carcinoma, Computed tomography

## Abstract

**Background:**

Mucinous tubular and spindle cell carcinoma of kidney (MTSCC-K) is a rare variant of renal tumor. The current data show most of MTSCCs are of low malignant potential and rare cases metastatic to lymph nodes have been reported; however, the recorded computed tomography (CT) and follow up data are limited.

**Material and method:**

In the present study, we retrospectively analyzed CT and clinicopathological data of eight patients with renal MTSCC-K.

**Results:**

A total of eight cases, including six females and two males, were included in this analysis with a mean age of 48.4 (range 25 to 81) years. Mean tumor size was 4.2 (range 2.5 to 10.0) cm. Preoperative CT demonstrated that all tumors were slightly enhanced on both corticomedullary and nephrographic phase, which was different from many other renal cell carcinomas. Three of them were treated with open radical nephrectomy, three with laparoscopic radical nephrectomy and the other two with laparoscopic partial nephrectomy. No postoperative therapy was applied. Patients were followed up for 15 to 64 months and there was no evidence of recurrence and metastasis.

**Conclusions:**

The MTSCC-K has special clinicopathological characteristics, low degree of malignancy and relative good prognosis. The diagnosis mainly depends on the histopathological examination and CT may help to differentiate with papillary renal cell carcinoma. Surgical treatment is recommended and additional therapies are not necessary.

**Virtual slides:**

The virtual slides for this article can be found here: http://www.diagnosticpathology.diagnomx.eu/vs/8435581771088249.

## Background

Mucinous tubular and spindle cell carcinoma of the kidney (MTSCC-K) is a rare pathological entity and has been described as a specific subtype of renal cell carcinoma (RCC) in the 2004 World Health Organization classification [1]. It is characteristically of elongated tubules lined by cords of spindle cells or cuboidal cells separated by mucinous extracellular matrix [2-4]. Though it was reported that MTSCCs showed a relatively good prognosis, the follow-up data is limited and the clinical behavior of this tumor remains to be established. And it’s necessary to gather more clinicopathological features of MTSCC-K for better diagnosis and treatment. Thus, we report our CT and clinicopathological findings of 8 MTSCC-K patients from January 2006 to December 2010 and relevant differential diagnoses are discussed.

## Case presentation

### Clinical data

Eight cases of MTSCC-K were identified from a review of approximately 1430 cases of RCC from the Department of Urology, Ren Ji Hospital between January 2006 and December 2010. Detailed clinical information of the 8 patients was listed in the Table 1. One patient was admitted to the hospital because of intermittent and painless gross hematuria lasted for 2 weeks, and another patient admitted for sufferring flank pain for almost half year. There were no symptoms of hematuria, flank pain or weight loss in the rest patients. There were no history of fever, hypertension, tuberculosis and diabetes. The patients’ general physical examination and routine blood test were within the normal range.

**Table 1:** Clinicopathological data of the patients with MTSCC-K

**Case No.**	**Sex**	**Age (years)**	**Size (cm)**	**TNM stage**	**Follow-up (months)**
1	M	37	6	T1bN0	64
2	FM	81	7.5	T1bNx	NA
3	FM	25	10.5	T2bNx	15
4	FM	59	5.5	T1bN0	34
5	FM	32	5	T1bN0	26
6	FM	60	7.5	T1bNx	60
7	M	34	2.5	T1aN0	64
8	FM	59	3.5	T1aN0	25

*M* Male, *F* Female, *NA* not available.

### Image findings

**Figure 1:**
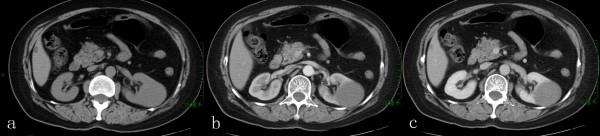
**Contrast enhanced abdominal CT scan.** It revealed a tumor on the middle portion of the kidney **(a)**, and demonstrated the tumor was slightly enhanced on the corticomedullary phase **(b)** and nephrographic phase **(c)**.

Hypovascular renal masses were noted in all cases on ultrasonography, and subsequent computed tomography was performed initially to obtain baseline attenuation values of lesions and to identify calcification; it demonstrated that all tumors had well-defined margins, and were slightly enhanced on both corticomedullary phase (CMP) and nephrographic phase (NP) (Figure 1a,b,c). The tumors’ attenuation values were ranged from 31 to 40 HU on non-contrast period; and 38 to 50 HU on CMP, 45 to 67 HU on NP, respectively (Table 2). Plain chest radiography and Emisson computed tomography were also preformed to verify there was no distant metastasis.

**Table 2:** CT Attenuation values (HU) of the patients with MTSCC-K

**Case No.**	**Non-contrast scan**	**CMP**	**NP**
1	35	43	63
2	40	50	57
3	NA	NA	NA
4	31	38	45
5	NA	NA	NA
6	36	45	54
7	37	50	67
8	NA	NA	NA

*MP* Corticomedullar phase, *NP* Nephrographicphase, *NA* not available.

### Treatment methods

Radical nephrectomy was applied to six cases, among which three were treated via laparoscopic approach. Laparoscopic partial nephrectomy was applied to another two cases as the tumors’ sizes were less than 4 cm. No postoperative therapy was given to any of the eight patients.

### Pathological findings

**Figure 2:**
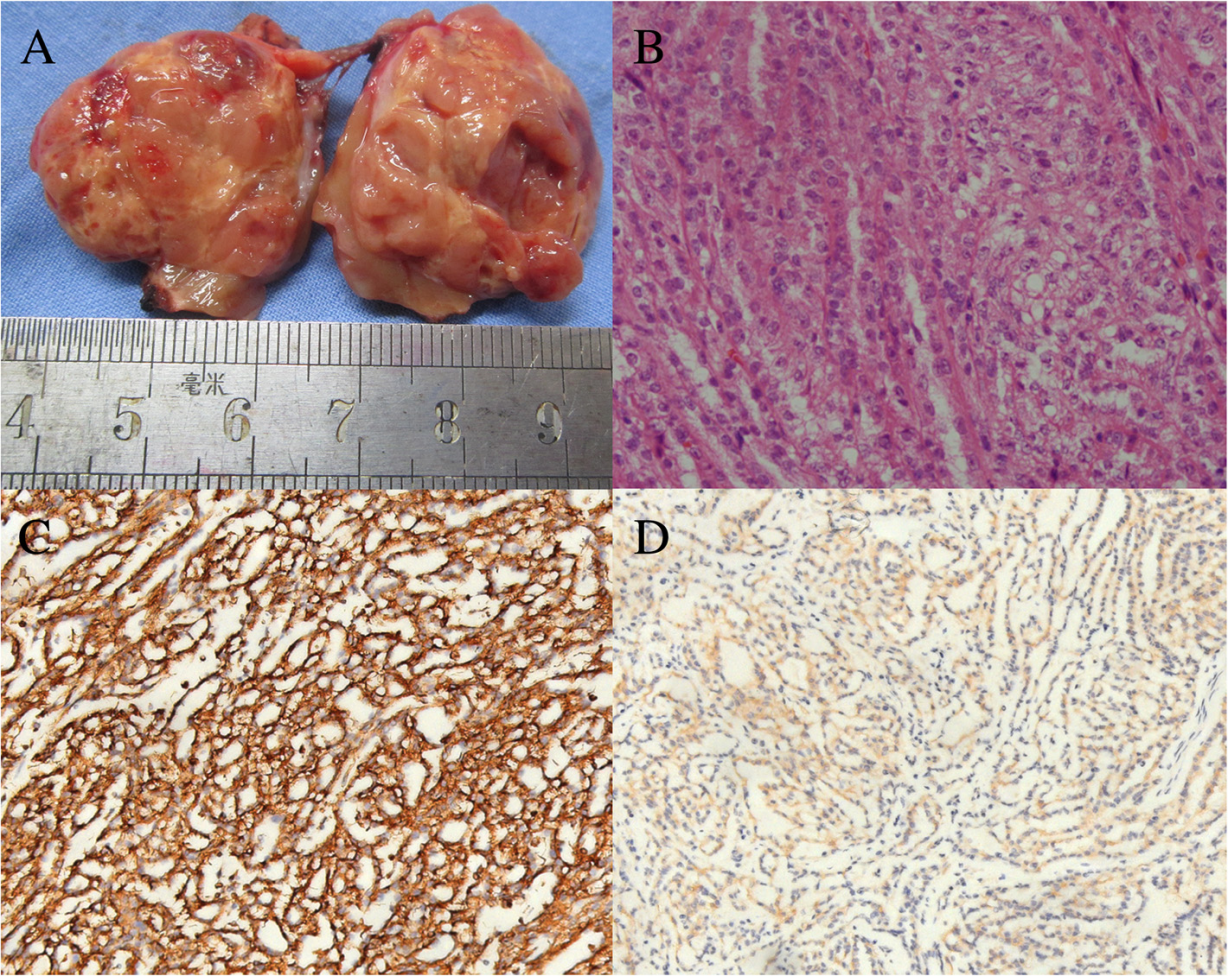
**Histopathological features of MTSCC-K.** Gross image of partial nephrectomy specimen displaying a small well-circumscribe solid grayish yellow tumor with a psuedocapsule **(A)**; microscopic findings shows the tumor cells composed of tubules seperated by mucinous stroma and a spindle cell component (**B**, original magnification × 400 HE); There is diffuse and strong immunohistochemical expression of Vimentin (**C**, ×200) in both tubules and spindle components and weak for VHL (**D**, ×200).

Grossly, these tumors were well-circumscribe solid, tan-yellow or grayish yellow in appearance (Figure 2A), with or without foci of hemorrhage or necrosis, the diameters varied between 2.5 and 10.5 cm. The surrounding perinephric fat, renal pelvis, and hilar vessels were identified and showed no involvement by tumor. Adrenal gland and lymph node metastasis were not detected. Histological examination of these tumors showed they were consisted of spindle or cuboidal cells arranged in tubular patterns embeded in mucinous or myxoid stroma (Figure 2B). However, the proportion of those components varied. Spindle cell areas were consisted of monotonous sheets of fairly uniform cells with large eosinophilic cytoplasm. The tubular pattern was made up of cuboidal cells with eosinophilic cytoplasm. Immunohistochemically, the tumors were strongly positive for AMACR (87.5%), EMA (37.5%), CK7 (62.5%), Vimentin (75%); and weak for VHL (45%) (Figure 2C, 2D).

## Discussion

Mucinous tubular and spindle cell carcinoma is a rare, malignant renal epithelial tumor which showed a female predominance and favorable prognosis and had been recognized as a new entity of RCC, and usually behaved in a low-grade fashion [1]. More than 80 cases have been reported and much is known about this tumor [5].

As described before, our results are similar to the previous studies. They, histologically, are characterized by small elongated tubules lined by cuboidal, spindle cells and variable amounts of myxoid stroma.

We further described the clinicopathological findings about the MTSCCs and pay particular attention to the CT features. On unenhanced scan, the MTSCC-K, as many of the other subtypes of solid RCCs, are with attenuation values ranged from 31 to 40 HU. And the attenuation values were ranged from 38 to 50 HU on CMP, 45 to 67 HU on NP, respectively. Many previous studies suggest that MTSCCs may represent a variant of papillary RCC [2,6], but the CT features are different of them. Post contrast CT image of papillary RCC in the CMP and NP shows uniform, mild enhancement approximately of 30 HU [7]. Zhang and his colleagues [8] suggested that low tumor-to-aorta enhancement ratio or tumor-to-normal renal parenchyma enhancement ratio was highly indicative of papillary RCC, which is consistent with the former findings [9]. However, in our series, the MTSCC-K show the tumor-to-normal renal parenchyma enhancement ratio was a little higher than 0.25. MTSCC-K as well as papillary RCC were typically less vascular compared with most other subtypes of RCC and most commonly manifested as homogeneous or peripheral enhancement. Noon [10] reported MRI characters of an incidentally detected case of MTSCC-K of an ectopic pregnancy patient; they calculated the T2 signal intensity ratio of MTSCC-K (0.96) using the method of Oliva et al. [11], which was different with papillary (0.67) and clear cell carcinoma (1.41).

In addition, MTSCC-K lack the gains of chromosomes 7 and 17 and losses of chromosome Y which are typical of papillary RCC [12], and using fluorescence in situ hybridization with centromeric probes for these chromosomes is helpful in differentiating papillary RCC from MTSCC-K. Besides, as Behnes et al. [13] reported, N-cadherin can be used to differentiate subtypes of papillary RCC and it might be a potential marker for differentiating MTSCC-K and papillary RCC.

Till now, the origin of MTSCC-K remains uncertain. In the literature, epithelial markers, especially AMACR, CK7, EMA and Vimentin, have been reported to be positive in 80-100% of cases [2], and these may support the hypothesis of a distal tubule origin. But AMACR could also be seen in the proximal convoluted tubules [6,14]. In our series, the tumors were strongly positive for AMACR (87.5%), CK7 (62.5%), Vimentin (75%); while only 37.5% showed strong EMA staining and 45% showed weak for VHL. However, immunohistochemical data do not enable the elucidation of the exact histogenenesis of MTSCC-K [2], and more special markers for its diagnosis need to be established in future.

In our series, prognosis seems to be favorable and there was no recurrence or metastasis, with a follow-up of between 15 and 64 months. However, occasional cases with local recurrence, distant metastasis and regional lymph node involvement have been reported [4,6,15,16]. In recent, 5 cases of sarcomatoid variant of MTSCCs with aggressive behavior have been reported in the literature, of which 3 cases of distant metastases with fatal outcome were seen [15-17]. It may suggest sarcomatoid changes are related to the bio-behavior of MTSCCs. And as few cases in published series have 5 or more years of follow-up, the true biologic potential and the morphologic criteria that define it remain unknown [18]. Thus, complete surgical resection is recommended and careful follow-up is necessary.

According to the literature and our series, the clinicopathological features of MTSCC are indicated as follows. Firstly, the tumor is characteristically consisted of large eosinophilic spindle or cuboidal cells arranged in tubular patterns embeded in mucinous or myxoid stroma [2]. Secondly, CT showed the tumor were slightly enhanced on both corticomedullary and nephrographic phase and the tumor-to-normal renal parenchyma enhancement ratio was a little higher than 0.25, which are different from other kinds of RCCs, together with MRI [10], it may help for the differentiation diagnosis of MTSCC. Thirdly, though the origin and exact histogenenesis of the tumor remain uncertain, using combination of immunohistochemistry may be helpful to the diagnosis of MTSCC [19] Last but not the least, most of MTSCC patients have no manifestations and prognosis seems to be favorable based on low histological grade, however, surgical resection and careful follow-up are recommended.

## Conclusion

The MTSCC-K has special clinicopathological characteristics, low degree of malignancy and relative good prognosis. Our reports are benifitial supplement for better understanding the clinicopathological features of MTSCC. Meanwhile, the CT findings, treatment and follow-up data are valuable information for guiding future clinical practice but further studies are required to confirm our computed tomography findings.

### Consent

Written informed consent was obtained from the patient for publication of this Case Report and any accompanying images. A copy of the written consent is available for review by the Editor-in-Chief of this journal.

## Abbreviations

MTSCC-K: Mucinous tubular and spindle cell carcinoma of the kidney; RCC: Renal cell carcinoma; CT: Computed tomography; CMP: Corticomedullary phase; NP: Nephrographic phase; HU: Hounsfield units.

## Competing interests

The authors declare that they have no competing interests.

## Authors’ contributions

X-RW and Y-HC designed the study, performed the histological and immunohistochemical evaluation, drafted the manuscript and performed the literature review; J-JS participated the literature review and collected the patient’s clinical information; LZ and J-WH carried out the immunohistochemical staining; J-JB and Y-RH participated in pathological investigations and helped to draft the manuscript .D-ML participated in pathological investigations, revised manuscript for important intellectual content and had given final approval of the version to be published. All authors have read and approved the final manuscript.

## References

[B1] Lopez-Beltran A Scarpelli M Montironi R Kirkali Z 2004 WHO classification of the renal tumors of the adults Eur Urol 2006 8 5 798 805 10.1016/j.eururo.2005.11.035 16442207

[B2] Ferlicot S Allory Y Compérat E Mege-Lechevalier F Dimet S Sibony M Couturier J Vieillefond A Mucinous tubular and spindle cell carcinoma: a report of 15 cases and a review of the literature Virchows Arch 2005 8 6 978 983 10.1007/s00428-005-0036-x 16231179

[B3] Kato M Soga N Arima K Sugimura Y A case of renal mucinous tubular and spindle cell carcinoma Int J Urol 2009 8 8 699 701 10.1111/j.1442-2042.2009.02332.x 19682107

[B4] Ursani NA Robertson AR Schieman SM Bainbridge T Srigley JR Mucinous tubular and spindle cell carcinoma of kidneywithout sarcomatoid change showing metastases to liver and retroperitoneal lymph node Hum pathol 2011 8 3 444 448 10.1016/j.humpath.2010.07.018 21194728

[B5] Yang G Breyer BN Weiss DA MacLennan GT Mucinous tubular and spindle cell carcinoma of the kidney J Urol 2010 8 2 738 739 10.1016/j.juro.2009.11.076 20022029 PMC3565602

[B6] Shen SS Ro JY Tamboli P Truong LD Zhai Q Jung SJ Tibbs RG Ordonez NG Ayala AG Mucinous tubular and spindle cell carcinoma of kidney is probably a variant of papillary renal cell carcinoma with spindle cell features Ann Diagn Pathol 2007 8 1 13 21 10.1016/j.anndiagpath.2006.09.005 17240302

[B7] Zhang J Lefkowitz RA Bach A Imaging of kidney cancer Radiol Clin North Am 2007 8 1 119 147 10.1016/j.rcl.2006.10.011 17157626

[B8] Zhang J Lefkowitz RA Ishill NM Wang L Moskowitz CS Russo P Eisenberg H Hricak H Solid renal cortical tumors: differentiation with CT Radiology 2007 8 2 494 504 10.1148/radiol.2442060927 17641370

[B9] Herts BR Coll DM Novick AC Obuchowski N Linnell G Wirth SL Baker ME Enhancement characteristics of papillary renal neoplasms revealed on triphasic helical CT of the kidneys AJR Am J Roentgenol 2002 8 2 367 372 10.2214/ajr.178.2.1780367 11804895

[B10] Noon AP Smith DJ McAndrew P Magnetic resonance imaging characterization of a mucinous tubular and spindle cell carcinoma of the kidney detected incidentally during an ectopic pregnancy Urology 2010 8 2 247 248 10.1016/j.urology.2009.09.047 19962725

[B11] Oliva MR Glickman JN Zou KH Teo SY Mortelé KJ Rocha MS Silverman SG Renal cell carcinoma: T1 and T2 signal intensity characteristics of papillary and clear cell types correlated with pathology AJR Am J Roentgenol 2009 8 6 1524 1530 10.2214/AJR.08.1727 19457814

[B12] Cossu-Rocca P Eble JN Delabunt B Zhang S Martignoni G Brunelli M Cheng L Renal mucinous tubular and spindle carcinoma lacks the gains of chromosomes 7 and 17 and losses of chromosome Y that are prevalent in papillary renal cell carcinoma Mod Pathol 2006 8 4 488 493 10.1038/modpathol.3800565 16554730

[B13] Behnes CL Hemmerlein B Strauss A Radzun HJ Bremmer F N-cadherin is differentially expressed in histological subtypes of papillary renal cell carcinoma Diagn Pathol 2012 8 95 10.1186/1746-1596-7-95 22888908 PMC3539962

[B14] Paner GP Srigley JR Radhakrishnan A Cohen C Skinnider BF Tickoo SK Young AN Amin MB Immunohistochemical analysis of mucinous tubular and spindle cell carcinoma and papillary renal cell carcinoma of the kidney: significant immunophenotypic overlap warrants diagnostic caution Am J Surg Pathol 2006 8 1 13 19 10.1097/01.pas.0000180443.94645.50 16330937

[B15] Dhillon J Amin MB Selbs E Turi GK Paner GP Reuter VE Mucinous tubular and spindle cell carcinoma of the kidney with sarcomatoid change Am J Surg Pathol 2009 8 1 44 49 10.1097/PAS.0b013e3181829ed5 18941398

[B16] Simon RA di Sant'agnese PA Palapattu GS Singer EA Candelario GD Huang J Yao JL Mucinous tubular and spindle cell carcinoma of the kidney with sarcomatoid differentiation Int J Clin Exp Pathol 2008 8 2 180 184 18784804 PMC2480554

[B17] Bulimbasic S Ljubanovic D Sima R Michal M Hes O Kuroda N Persec Z Aggressive high-grade mucinous tubular and spindle cell carcinoma Hum pathol 2009 8 6 906 907 10.1016/j.humpath.2009.03.004 19442792

[B18] Fine SW Argani P DeMarzo AM Delahunt B Sebo TJ Reuter VE Epstein JI Expanding the histologic spectrum of mucinous tubular and spindle cell carcinoma of the kidney Am J Surg Pathol 2006 8 12 1554 1560 10.1097/01.pas.0000213271.15221.e3 17122511

[B19] Liu Y Qiu XS Wang EH Sporadic hemangioblastoma of the kidney: a rare renal tumor Diagn Pathol 2012 8 49 10.1186/1746-1596-7-49 22548972 PMC3488519

